# PLOS Biology and the life sciences in 2024

**DOI:** 10.1371/journal.pbio.3002985

**Published:** 2024-12-18

**Authors:** Daniel Routledge, on behalf of the PLOS Biology Staff Editors

**Affiliations:** Public Library of Science, San Francisco, California, United States of America, and Cambridge, United Kingdom

## Abstract

As we reach the end of 2024, our Editorial celebrates an amazing year of science for PLOS Biology and the life sciences more broadly, and thanks everyone who supports the journal.

## Teaser

As we reach the end of 2024, we celebrate an amazing year of science for PLOS Biology and the life sciences more broadly, and thank everyone who continues to support the journal.

Phew. We made it. The end of 2024 is here, and what a year it’s been. As ever, science has been at the forefront of news stories this year. Despite increasing science scepticism and anti-science sentiment in the media and public, we hope that fact-based decision-making will prevail in 2025 and beyond. As 2024 is set to be the hottest year on record, the effects of climate change are becoming increasingly evident: We have seen devastating extreme weather events across the globe, from Hurricane Milton in the US, to floods across Europe and Asia, and “exceptional” wildfires in the Americas. But 2024 is also a year to celebrate steps in the right direction in our fight against climate change, as rates of deforestation in the Brazilian Amazon decreased 31% compared to 2023, and in the first half of 2024, wind and solar power generated more electricity in the EU than fossil fuels for the first time.

Elsewhere in the life sciences, we’ve gained insights into our evolutionary past with the first evidence of two hominins walking side by side [1], world-firsts in stem cell therapy, e.g. for the treatment (reversal) of type I diabetes [2], and the launch of AlphaFold3, which will revolutionize structural biology research with its ability to predict not only protein structures, but also interactions with other molecules such as DNA and RNA [3]. Quite aptly, the 2024 Nobel Prize in Chemistry was awarded to Demis Hassibis and John Jumper, the developers of AlphaFold, and shared with David Baker for his work on computational protein design. Alongside the Nobel Prize in Physics for the development of machine learning in artificial networks, these are the first Nobel prizes awarded to applications of artificial intelligence, perhaps an indication of the future direction of science.

At *PLOS Biology*, we are also celebrating a great year of science, in which we have published more research articles than ever. We’ve put together a list of our editors’ recommendations from 2024 (Box 1), so when you’re sat at home between Christmas and New Year’s wondering what day it is, or need an escape from hosting the in-laws, you need look no further for some captivating science to escape to. We also published three special collections of magazine articles on ‘Symbiosis across the tree of life’, ‘Bringing data to decision making for conservation and biodiversity’, and ‘The promises and challenges of neurotechnology to improve human health and cognition’, bringing attention to these diverse yet equally important topics. And if you want to keep up to date with what we publish in 2025, be sure to follow our social media channels (BlueSky: @plosbiology.org; Mastodon: @PLOSBiology@fediscience.org).

With the growing success of the journal, this year we expanded our editorial team, and we welcomed Taylor Hart as an Associate Editor for neuroscience and behavioral ecology to the *PLOS Biology* family. We also expanded our editorial family, welcoming three new PLOS Biology babies, and had the pleasure of working with Suzanne de Bruijn for six months while she covered two parental leaves. Suzanne actually started her editorial career with *PLOS Biology* as an intern in 2022 and covered her experience in a Biologue post. I have also had the pleasure of joining the journal to cover the maternity leave of our Front Section Senior Editor, Joanna Clarke, to whom we bid a temporary farewell.

**Box 1 – PLOS Biology editors’ top picks for 2024**

**Nonia Pariente, Editor-in-chief**

My choice is “Deep mutational scanning of H5 hemagglutinin to inform influenza virus surveillance” by Dadonaite et al., [4]. This systematic study of the effects of mutations can inform real-time interpretation of viral variation observed during surveillance of H5 influenza, which is of particular value given the ongoing broad circulation in mammals and birds worldwide, including US cattle farms.

**Ines Alvarez-Garcia, Section Manager** (cell biology, signaling, development, aging, cancer and plant biology)

My choice is “Transcriptomic analysis of the 12 major human breast cell types reveals mechanisms of cell and tissue function” by Del Toro et al., [5]. These authors perform a transcriptomic analysis of the 12 major cell types of the human breast and provide an invaluable resource to study cell-intrinsic characteristics and human breast tissue composition. This information is crucial to understanding breast cancer and other pathologies.

**Roli Roberts, Section Manager** (evolutionary biology, ecology and meta-research)

My choice is “Single-fly genome assemblies fill major phylogenomic gaps across the Drosophilidae Tree of Life” by Kim et al., [6]. Our genomic view of the Tree of Life of small organisms has been restricted by our ability to grow them in the lab in sufficient numbers to generate large amounts of DNA. These authors get around this problem in the case of the Drosophilidae, a large family of tiny fruit flies. The authors generate new genome assemblies for 179 species of drosophilids, but incredibly, for 121 of these species, the assembly is based on DNA from a single individual fly.

**Christian Schnell, Section Manager** (neuroscience)

My choice is “Selective suppression of oligodendrocyte-derived amyloid beta rescues neuronal dysfunction in Alzheimer’s disease” by Rajani et al., [7]. This paper is one of a few papers this year that reveal the importance of oligodendrocytes in Alzheimer’s disease. This type of glia cells has received relatively little attention in the context of many neurodegenerative diseases, so it’s interesting to know that they apparently contribute actively to the pathology as well.

**Daniel Routledge, Front Section Senior Editor**

My choice is “A new lineage nomenclature to aid genomic surveillance of dengue virus” by Hill et al., [8]. In this Consensus View, the authors propose a new lineage nomenclature system for dengue virus (DENV) that will facilitate better classification and monitoring of DENV sub-lineages. Given the increasing genetic diversity and prevalence of DENV, this work is crucial in ensuring this diversity is effectively identified and monitored. This updated classification system will therefore be important for informing public health authorities and their efforts to track and prevent DENV transmission.

**Richard Hodge, Senior Editor** (molecular biology, genetics/genomics, cancer, structural biology, systems biology, methods and biotechnology)

My choice is “The extracellular matrix supports breast cancer cell growth under amino acid starvation by promoting tyrosine catabolism” by Nazemi et al., [9]. Here, the authors studied how extracellular matrix in the tumor microenvironment supports breast cancer growth. They found that invasive breast cancer cells internalize extracellular matrix components via macropinocytosis, providing a source of amino acids via phenylalanine and tyrosine metabolism.

**Lucas Smith, Senior Editor** (neurodegeneration, physiology and disease, metabolism, stem cell biology, epigenetics and circadian rhythms)

My choice is “Neuronal cell cycle reentry events in the aging brain are more prevalent in neurodegeneration and lead to cellular senescence” by Wu et al., [10]. During aging there is evidence that a small number of terminally differentiated neurons can upregulate genes related to the cell cycle. This study has developed a potentially broadly useful approach to identify and characterize these cells in human and mouse snRNA-seq datasets - and they use this approach to provide interesting insights into the prevalence and fate of these neurons in aging and neurodegeneration.

**Taylor Hart, Associate Editor** (neuroscience and behavior)

My choice is "An evolutionarily conserved pathway mediated by neuroparsin-A regulates reproductive plasticity in ants" by Zhang et al., [11]. In social insects, events such as mating or a depletion in certain pheromones can cause females with reproductive potential to express queen-like traits. In this study, the authors show that, in two ant species, a conserved neuropeptide suppresses reproduction and induces worker-like behavioral patterns. This study provides another piece in the puzzle of how social insects evolved to use ancestral neurohormone pathways in new ways to support polyphenism related to caste and reproductive status.

**Melissa Vazquez Hernandez, Associate Editor** (microbiology and immunology)

My choice is “Multicellular magnetotactic bacteria are genetically heterogeneous consortia with metabolically differentiated cells" by Schaible et al., [12], which explores the complex lifestyle of multicellular magnetotactic bacteria— the only known bacteria that entirely lack a unicellular stage in their life cycle. The study shows that these consortia are formed of genetically diverse cells that exhibit a clear division of labor. These findings not only highlight the complexity of these unique organisms but also expand our understanding of the diverse strategies that bacteria have developed to thrive in various environments.

But our Staff Editors could not do the work they do without a little (a lot of) help; we would like to thank all of the Academic Editors for their invaluable expertise and advice, as well as the staff behind the scenes who keep the journal running. We would also like to thank all the authors, reviewers, and you, the reader, for continuing to support *PLOS Biology* and Open Access publishing through 2024. So, raise a glass (or conical flask, Eppendorf, petri dish… your vessel of choice) to a remarkable 2024, and to an equally fruitful 2025.
